# Deep learning for automated materials characterisation in core-loss electron energy loss spectroscopy

**DOI:** 10.1038/s41598-023-40943-7

**Published:** 2023-08-22

**Authors:** Arno Annys, Daen Jannis, Johan Verbeeck

**Affiliations:** 1 EMAT, University of Antwerp, 2020 Antwerp, Belgium; 2 Nano center of excellence, University of Antwerp, 2020 Antwerp, Belgium

**Keywords:** Characterization and analytical techniques, Characterization and analytical techniques

## Abstract

Electron energy loss spectroscopy (EELS) is a well established technique in electron microscopy that yields information on the elemental content of a sample in a very direct manner. One of the persisting limitations of EELS is the requirement for manual identification of core-loss edges and their corresponding elements. This can be especially bothersome in spectrum imaging, where a large amount of spectra are recorded when spatially scanning over a sample area. This paper introduces a synthetic dataset with 736,000 labeled EELS spectra, computed from available generalized oscillator strength tables, that represents 107 K, L, M or N core-loss edges and 80 chemical elements. Generic lifetime broadened peaks are used to mimic the fine structure due to band structure effects present in experimental core-loss edges. The proposed dataset is used to train and evaluate a series of neural network architectures, being a multilayer perceptron, a convolutional neural network, a U-Net, a residual neural network, a vision transformer and a compact convolutional transformer. An ensemble of neural networks is used to further increase performance. The ensemble network is used to demonstrate fully automated elemental mapping in a spectrum image, both by directly mapping the predicted elemental content and by using the predicted content as input for a physical model-based mapping.

## Introduction

Electron energy loss spectroscopy (EELS) is an analytical technique in (scanning) transmission electron microscopy ((S)TEM) that yields information on the elemental content of a sample in a very direct manner. The energy at which a core-loss edge appears reveals what element-specific ionization occurred. EELS in STEM yields large amounts of data in the form of spectral images, which allow a mapping of the spatial distribution of elements in a sample at atomic scale. EELS spectra recorded with a sufficient energy-resolution reveal, through the fine structure of a core-loss edge, information on the electronic state of the sample, like oxidation state and bonding. Besides a qualitative study of EELS spectra, like element identification and mapping, a quantitative study allows to determine the relative and absolute quantities of elements in the sample. Model-based approaches^1^ to quantification require as input the elemental content of the spectrum. Therefore, it is currently still common-practice for an expert to perform time-consuming visual inspections of EELS spectra for element identification.

Early attempts at automated identification and quantification of core-loss edges relied on the use of filter-based methods and tabulated edge properties, especially the edge onset energy^2,3^. These methods had limited success in real-world applications due to their high noise sensitivity and difficulty in dealing with low jump-ratio edges. In the current age of artificial intelligence, data-driven methods, especially deep learning methods which use neural networks (NN), are capable of solving a large number of tasks, given that enough training data is supplied. Unsupervised techniques, like K-means clustering^4^, non-negative matrix factorization^5,6^ and auto-encoders^7^ have been extensively applied in EELS for spectral decomposition. Supervised techniques, like NN and support vector machines, allow for more generic EELS applications like oxidation state determination^8–10^, zero-loss peak determination^11^, spectral deconvolution^12^ and phase-transition forecasting^13^. NN have also been successfully applied in many techniques similar to EELS like X-ray diffraction^14^, vibrational spectroscopy^15^, X-ray fluorescence spectroscopy^16^, energy-dispersive X-ray spectroscopy^17^ and molecular excitation spectroscopy^18^. Deep learning was only recently applied for element identification in EELS by Kong et al.^19^, who developed a synthetic dataset containing K or L core-loss edges for 20 common elements. Their simulation method relied on the processing of experimental data. Core-loss edges from experimental spectra were extracted by multi-Gaussian fitting and adapted by means of scaling, shifting and noising. Multiple edges and a background were then combined to form a synthetic spectrum. The main limitation of this approach is the limited amount of experimental data that is available, especially for heavier elements. Multiple elements in their dataset, like e.g. S and Cl, are represented by only a single experimental edge in the dataset formation. A lack of variation in training data limits NN in generalizing effectively when facing new data. Furthermore, their simulation method allowed only one fixed energy loss range, which severely reduces the practical applicability.

This paper proposes a purely computational EELS dataset, consisting of 736,000 spectra, based on available generalised oscillator strength (GOS) tables. The dataset represents 107 K, L, M or N core-loss edges and 80 chemical elements. The dataset supplies ground-truth labels for both element identification and relative quantification. Our simulated dataset has a flexible detector range thanks to zero padding of the spectra to a wide detector range of 0 to 3071 eV. Multiple NN architectures—being a multilayer perceptron, a convolutional neural network, a U-Net, a residual neural network, a vision transformer, a compact convolutional transformer and an ensemble—are optimized and evaluated in terms of efficiency and performance for element identification on both simulated and experimental data.

## Methods

### Synthetic dataset formation

#### Specimen sampling

**Figure 1:**
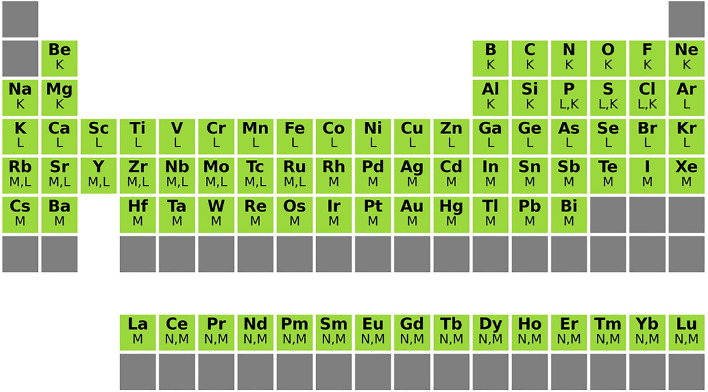
80 elements and respective 107 edges represented in the simulated dataset.

Dataset-formation methods based on experimental data are constrained by the limited amount of labeled data, especially for heavier elements. A completely computational dataset has the advantage of allowing a much broader set of elements and edges. Figure 1 shows the elements and respective edges that are included in our presented dataset. H, He and Li are excluded because they do not have edges in the core-loss region. Elements heavier than Bi are excluded because they are rare. Instead of forming samples through arbitrary combinations of elements, samples are drawn from the list of materials on The Materials Project^20^. For each element included in the dataset, a list of samples containing at least this element is drawn from The Materials Project. As a result of this approach, each element will fulfill a minimal occurrence in the dataset, but common elements will be more present in the dataset because they often occur as an additional element in a sample, next to the query element. Given the extreme differences in natural occurrence of elements, this approach compromises between biasing the dataset and losing all prior-knowledge. The training set contains, for each element, 1400 samples and 5 spectra for each sample. The test set and validation set each contain 300 samples for each element and respectively 5 and 2 spectra for each sample. An additional dataset is simulated where each spectrum contains only core-loss edges corresponding to a single element. This can be used to determine what pairs of elements are often confused.

#### Spectrum simulation

As can be seen in Fig. 1, some elements in the dataset are represented by two sets of edges. A spectrum could contain either a single one of these two sets, or both sets together. Since only a single set of edges should be sufficient in order to be able to identify an element, we allow the possibility for one of the two sets to fall outside of the detector range. A random detector range is chosen, using only the constraints that the query element must have at least one set of major edges within the detector range. This ensures that the minimal occurrence of each element is maintained like it is in the sample formation. The edges for the additional elements in the sample are included if they fall within the random detector range, meaning that the spectrum starts at least 10 eV before the edge onset and goes on for at least 25 eV beyond the edge onset. The content of a spectrum can thus—like it does in practice—differ from the general content of the sample depending on the chosen detector range.

**Figure 2:**
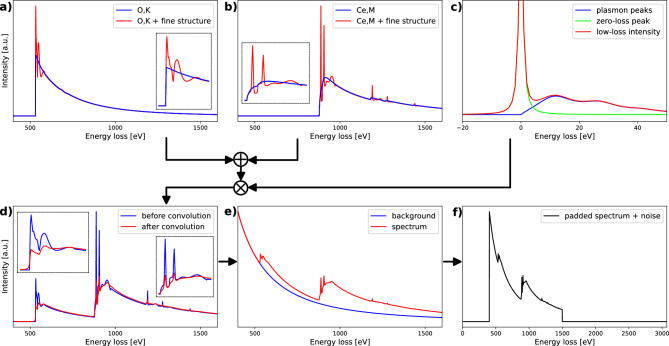
Diagram of the simulation of a ${\text{CeO}}_{2}$ spectrum. (**a**) and (**b**) Core-loss edges are calculated from GOS tables and given a generic fine structure. (**c**) A generic low-loss region is simulated. (**d**) The summed core-loss edges are convolved by a generic low-loss region. (**e**) A power law background is added. (**f**) The final spectrum is normalised, zero padded and Poisson noise is applied.

The theory of inelastic electron scattering, which forms the foundation of the simulation of EELS spectra, has been extensively described by Egerton^21^. Our spectrum simulation method relies on publicly available GOS tables^22^ and edge onset energies from the EELS atlas^23^. The calculation of a core-loss edge requires a series of parameters like the microscope’s acceleration voltage, beam convergence angle and collection angle. These parameters are randomly drawn from uniform distributions for each individual spectrum simulation. Additionally, a random chemical shift is applied to the onset energy of each edge. Since the available GOS tables do not consider solid-state effects, resulting core-loss edges do not portray fine structure. Calculating the fine structure for each sample in the dataset is extremely computationally expensive. Therefore, generic fine structures are used instead, which do not represent the true fine structure of a given edge in a specific material but do show a sufficiently similar profile. Such a generic fine structure is formed through a sum of randomly weighted Gaussian peaks, occurring at random energy losses in proximity to the onset energy, with lifetime broadened widths. As described by Egerton^21^, the lifetime of the ionized atomic electron can be estimated as $\tau _f \approx \lambda _i / \nu$ where $\lambda _i$ is the inelastic mean free path and $\nu =\sqrt{2 \varepsilon /m}$ is the velocity of the ionized electron, with $\varepsilon$ the energy loss above the edge onset. The width of the lifetime broadened Gaussian peaks is then determined from Heisenberg’s uncertainty principle $\Gamma _f \approx \hbar / \tau _f$. This method requires the electron’s inelastic mean free path, which was parameterised by Seah and Dench^24^ for solids consisting of one element as $\lambda _i= 538a\varepsilon ^{-2} + 0.41 a^{3/2} \varepsilon ^{1/2}$, where $\lambda _i$ and the atomic diameter *a* are expressed in nanometers. Since the atomic diameter within a sample is unknown, it is randomly sampled between 25 and 250 pm. Additional random parameters include the degree of the fine structure, i.e. the number of contributing Gaussian peaks and the width of the fine structure, i.e. the energy range above the edge onset in which fine structure occurs. The relative scale of the fine structure with respect to the calculated core-loss edge is made to depend on the fine structure’s ratio of most positive value to most negative value. The largest allowed ratio of fine structure peak amplitude to edge peak amplitude is 10. This simulates the effect of strong white lines. Similar to the fine structure approach, a generic low-loss region is simulated by a Lorentzian zero-loss peak with an arbitrary width plus an arbitrary number of plasmon peaks, simulated by the Drude model, with an arbitrary energy and width. The zero-loss peak and plasmon peaks are scaled by the probability $P_n$ that an electron suffers n collisions, which under the assumption that each scattering event is independent corresponds to a Poisson process where $P_n = \frac{1}{n!} \left( \frac{t}{\lambda } \right) ^n \exp (-\frac{t}{\lambda })$. The scattering parameter $\frac{t}{\lambda }$ is also drawn from a uniform distribution. All core-loss edges are added and the sum is convolved with the low-loss region to simulate the effect of multiple scattering. This convolution tends to significantly decrease the amplitude of simulated white lines. An $A \left( E / E_0 \right) ^{-r}$ background is added, where *A* and *r* are randomly drawn and $E_0$ is the starting energy of the spectrum, which had also been randomly drawn. A jump-ratio—defined as the ratio of the peak amplitude of the core-loss edge before fine structure to the amplitude of the background at the edge onset—between 0.2 and 1.5 is enforced on one arbitrarily chosen edge. This directly determines the jump-ratio of all other edges in the spectrum, which can be boundlessly small or large. Poisson noise is applied and the signal to noise ratio is fully determined by the previous parameters, the background amplitude *A* in particular. A random instrumental shift—which in practice might result from misalignment or drift of the microscope or spectrometer components—is applied. Finally the spectrum is normalized and zero padded so that all spectra are of the required input shape for a NN. Table 1 shows the values of the parameters used for the simulation, or the distributions from which they are drawn. Figure 2 shows a schematic overview of the simulation procedure for ${\text{CeO}}_{2}$.

**Table 1:** Parameters of the spectrum simulation.

Parameter	Value
Acceleration voltage	$\in [60, 100, 200, 300]$ kV
Convergence angle	$\in {\mathbb {R}} \text {; } \in [1, 20]$ mrad
Collection angle	$\in {\mathbb {R}} \text {; } \in [1, 100]$ mrad
Energy dispersion	1 eV
Spectrum start	$\in {\mathbb {N}} \text {; } \in [75, E_{first \, edge}-10]$ eV
Spectrum end	$\in {\mathbb {N}} \text {; } \in [E_{last \, edge}+25, 3272]$ eV
Spectrum range (zero padding)	0 to 3071 eV
FWHM of zero-loss peak	$\in {\mathbb {R}} \text {; } \in [1,3]$ eV
Plasmon energy	$\in {\mathbb {N}} \text {; } \in [3,20]$ eV
Plasmon width	$\in {\mathbb {N}} \text {; } \in [3,20]$ eV
Number of plasmon peaks	$\in {\mathbb {N}} \text {; } \in [2,5]$
Scattering parameter $\frac{t}{\lambda }$	$\in {\mathbb {R}} \text {; } \in [0.1,1]$
Jump-ratio	$\in {\mathbb {R}} \text {; }\in [0.2,1.5]$
Background exponent	$\in {\mathbb {R}} \text {; }\in [2.0,4.0]$
Background amplitude	$\in [10^3,10^4,10^5,10^6,10^7]$
Fine structure width	$\in {\mathbb {N}} \text {; } \in [50,100]$ eV
Fine structure degree	$\in {\mathbb {N}} \text {; } \in [10,25]$
Maximum fine structure scale	10
Atomic diameter	$\in {\mathbb {R}} \text {; } \in [25,250]$ pm
Chemical shift	$\in {\mathbb {Z}} \text {; } \in [-5,5]$ eV
Instrumental shift	$\in {\mathbb {Z}} \text {; } \in [-5,5]$ eV

### Experimental dataset

**Figure 3:**
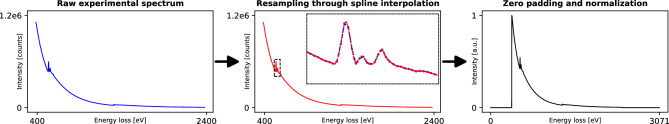
Pre-processing procedure for experimental spectra. A spline is used to resample the spectrum to the required energy axis and the resulting spectrum is zero padded and normalized.

An experimental dataset containing 279 element occurrences in 197 spectra—which are completely independent from the simulated training dataset—is formed using data from the Electron Energy Loss Data Center (EELDC)^25^, Gatan’s EELS Atlas^23^ and the Electron Energy-Loss Spectroscopy and X-Ray Absorption Spectroscopy Database (EELSDB)^26^. Some spectra gathered from the EELSDB have citation information available:^27–35^. Pre-processing is kept as minimal as possible to ensure a fast flow of recorded spectra to the NN, enabling real-time predictions at the microscope. The pre-processing procedure is shown in Fig. 3, where an experimental spectrum is resampled to the required energy axis, zero padded to the full energy loss range and normalized.

**Figure 4:**
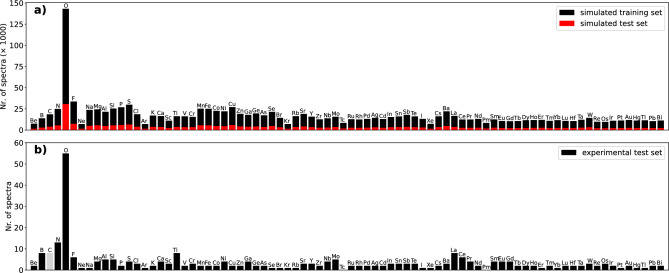
Occurrence of each element in (**a**) simulated training and test dataset (**b**) experimental dataset. Carbon’s occurrence in experimental spectra is ambiguous due to it’s common presence in the sample grid.

Figure 4 shows the occurrence of each element in both the simulated and the experimental datasets. Carbon’s occurrence in the experimental spectra is ambiguous due to it’s common presence in the sample grid. As expected, there is a noticeable similarity between the occurrence of elements in the simulated and experimental datasets. The experimental dataset is randomly split into a validation set with 97 spectra and test set with 100 spectra.

### Element identification models

Since it is a priory unknown what NN architectures perform best for a given task on a given dataset, 6 NN architectures are compared in terms of efficiency and performance. The compared architectures are a multilayer perceptron (MLP), a convolutional NN (CNN), a U-Net^36^, a residual NN (ResNet)^37^ and 2 transformer networks^38^ being a vision transformer (ViT)^39^ and a compact convolutional transformer (CTT)^40^. The MLP is known to no longer be state-of-the-art in computer vision, amongst other reasons because it is not able to learn translation invariance as well as CNN. For element identification in EELS, there is limited need for such translation invariance, on the contrary, the onset energy is the most important form of information. Therefore, it is interesting still to consider the MLP. CNN—because of their immense importance in computer vision—come in many configurations. The CNN chosen to be evaluated is a one dimensional version of the well-known VGG-11 network^41^. The U-Net architecture—which was originally introduced as an image segmentation CNN—has proven very successful due to its ability to combine global and local contextual information. To form a classification prediction using the U-Net, a MLP classification head is appended. ResNets were successfully introduced to tackle the vanishing gradient problem in very deep CNN. A 35 layer deep ResNet is evaluated. Kong et al. have proposed a convolutional-bidirectional long short-term memory NN (CNN-BiLSTM) for element identification in EELS spectra. However, the LSTM being a recurrent neural network (RNN) has the issue of being particularly slow due to the constraint to sequential computation. RNN and the LSTM are rapidly being replaced by attention-based architectures like transformers. The transformer was originally introduced as a natural language processing model, but has also known great success in computer vision with i.a. the ViT. The ViT divides it’s input into patches that are encoded prior to being processed by a transformer encoder. The CCT was introduced to increase the efficiency of the ViT and replaces partitioning in to patches by convolutional layers. The CTT’s design has many similarities to that of the CNN-BiLSTM. Additionally, an ensemble of models—which is a common technique used to improve robustness and accuracy in exchange for a longer inference time^42^—is compared. In the ensemble network, the predictions of multiple models—which could be different instances of either the same or different architectures—are averaged. The constituents of the ensemble network are chosen depending on the performance of the above described architectures. We choose to limit the size of the ensemble network to one that can calculate inside the working memory of a NVIDIA GeForce RTX 3060 GPU, as if it were a single NN.

**Figure 5:**
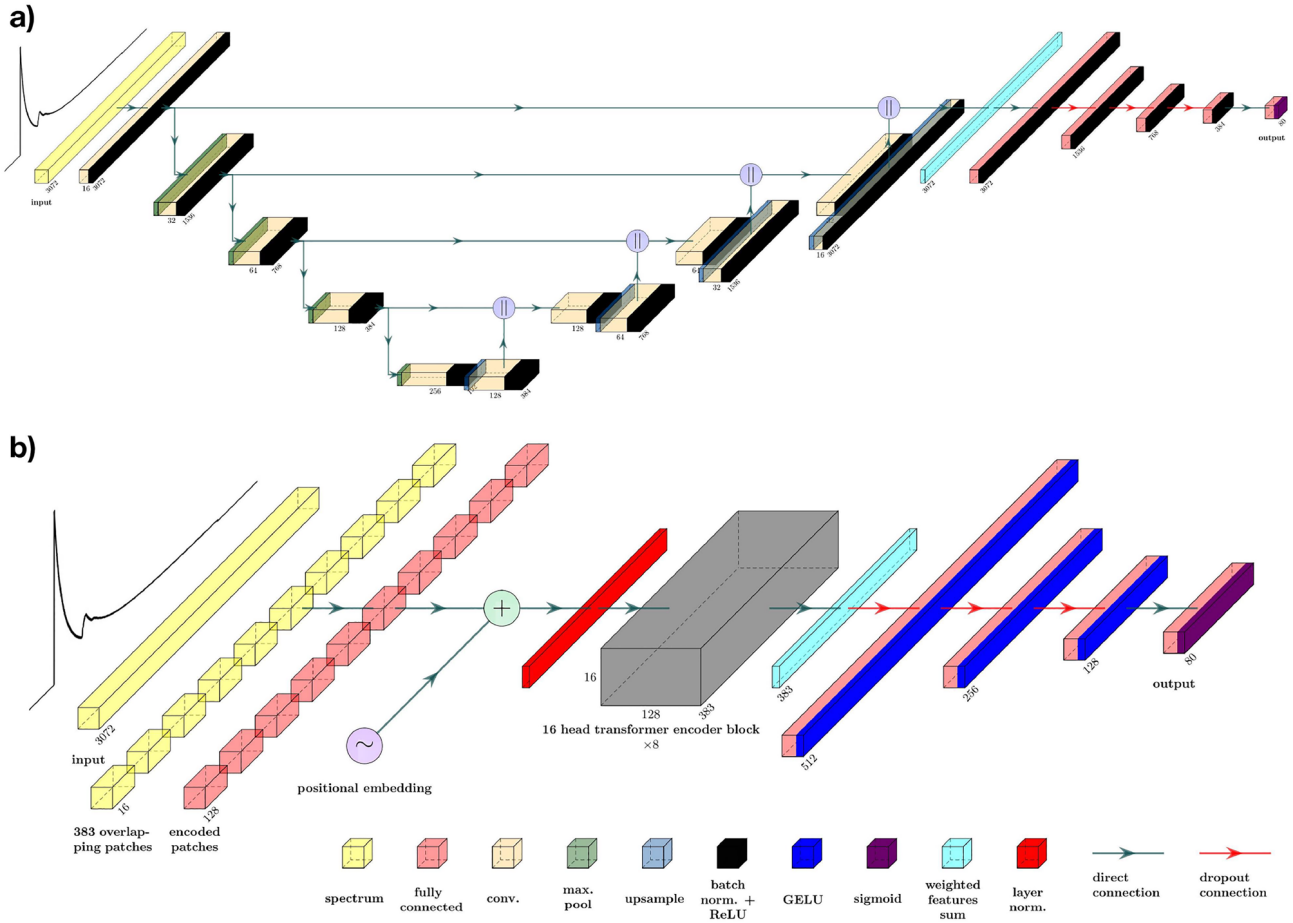
Schematic overview of (**a**) the U-Net architecture (**b**) the ViT architecture. Visualization made using software adapted from PlotNeuralNet^44^.

The original ResNet and ViT architectures use global average pooling (GAP) over the spatial dimensions so that feature maps can be passed to the MLP classification head. To append a MLP classification head to the U-Net architecture, some dimension reduction method must be applied as well. GAP of the energy axis suppresses crucial information for element identification. Alternatives to GAP are a learnable weighted sum of features, which is used in the original U-Net, or flattening, which has a high parameter cost. The ViT is also often used with a class token method and the CCT was introduced with the sequence pooling method, which also suppresses the spatial information. The best approach for each architecture is determined experimentally. The activation functions from the original architectures are maintained, except for the output layer. Since the task at hand is a multi-class and multi-label problem, one cannot use the common softmax activation function. Instead, sigmoid activation is used in all output layers, so that the model’s prediction can be interpreted as the confidence that an element is present. Either batch normalization or layer normalization are applied in all models. Figure 5 shows a schematic overview of the used U-Net and ViT architectures. Schematics for the remaining architectures are presented in the additional information (Supplementary Figures).

The NN are evaluated by precision, recall, and their harmonic mean, known as the F$_1$-score. These metrics are weighted by the occurrence of each element in the dataset. Furthermore, the evaluation includes the exact match rate (EMR), which measures the percentage of spectra where the predicted content exactly matches the true content, and the root mean square error (RMSE). All NN are trained using the Adam optimizer^43^ and a custom loss function which is the sum of the binary cross-entropy loss function and a macro soft F$_1$ loss function. For each model the initial learning rate is optimized by a grid search, and the learning rate is halved on plateaus of the F$_1$-score to increase convergence. When calculating the metrics for experimental data, the network’s predictions for carbon are not taken into account due to the ambiguity of the ground truth label. Since the task at hand is a multi-class and multi-label problem, the network’s output must be thresholded in order to be able to compute these metrics. The optimal threshold depends on the application and preference of the user. In what remains, the applied threshold is the one that minimizes the difference between precision and recall in the validation data.

## Results and discussion

### Element identification

The threshold for experimental data is noticeably higher than the threshold for simulated data, likely due to the many core-loss edges with boundlessly small jump-ratios in the simulated spectra. Table 2 compares the F$_1$-score, EMR and RMSE achieved by each model on both the simulated and experimental test set, utilizing the optimal threshold determined respectively on the simulated and experimental validation set. Despite it’s large number of parameters, the MLP demonstrates poor performance. While the CNN and ResNet exhibit good performance on simulated data, they struggle to generalize effectively to experimental data, potentially attributable to their high number of trainable parameters. Only the U-Net and the ViT manage to achieve both an F$_1$-score exceeding 80% and an EMR of at least 60% on the experimental data. The CCT is generalizing better than the CCN and ResNet, but poorer than the ViT and U-Net.

**Table 2:** Comparison of the identification model architectures.

Model	# of parameters [mln.]	Simulated test set			Experimental test set			Inference time [s]
		F$_1$-score	EMR	RMSE	F$_1$-score	EMR	RMSE	
MLP	56	0.50	0.05	0.16	0.42	0.12	0.15	0.1
CNN	45	0.90	0.68	0.07	0.76	0.42	0.12	0.2
ResNet	41	0.89	0.68	0.07	0.77	0.53	0.10	0.3
U-Net	20	0.86	0.63	0.07	0.84	0.62	0.09	0.2
ViT	2	0.84	0.55	0.09	0.84	0.60	0.08	0.6
CCT	5	0.87	0.60	0.08	0.79	0.58	0.08	0.6

Table 3 shows a more detailed evaluation of the ViT and U-Net, alongside an evaluation of an ensemble network consisting of two ViTs and three U-Nets. Note that the F$_1$-score must not be in between precision and recall because weighted averaging is used.

**Table 3:** Evaluation of ViT, U-Net and ensemble model on the experimental test set.

Model	F$_1$-score	Precision	Recall	EMR	Threshold
ViT	0.84	0.86	0.86	0.60	0.80
U-Net	0.84	0.86	0.86	0.62	0.95
2$\times$ViT+3$\times$U-Net	0.86	0.88	0.88	0.70	0.75

**Figure 6:**
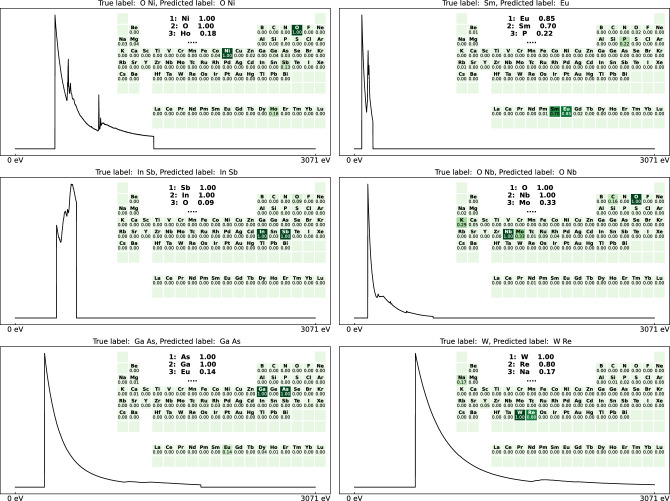
Examples of experimental spectra with predictions by the 2$\times$ViT+3$\times$U-Net ensemble.

Interpretation of these results is hindered by the absence of comparable results. The accuracy of a human expert has not been quantified and the task described by Kong et al. is not directly comparable. The EMR of 70% could at first glance be considered rather unsuccessful, but one should not forget that EMR is a very demanding metric. A model with an EMR of 70% can not be interpreted as a model that only yields useful predictions 70% of the time. Precision and recall both being 88% implies that a present element has an 88% chance of being detected and a detected element has an 88% chance of actually being present. A confusion matrix that quantifies pairs of elements that are often confused by the ensemble network is given in the additional information (Supplementary Figures). Figure 6 shows six examples of experimental spectra with predictions by the ensemble network. These examples demonstrate how the network can correctly process spectra within a wide range of characteristics, for example ranging from minimal fine structure to strong white lines or ranging from broad to localised energy loss regions. The examples show two typical cases of mistaken predictions. First, the N edges for the neighbouring lanthanides often have differences in onset energies smaller than the variations due to chemical and instrumental shifts, causing confusion between neighbouring lanthanides. The second common mistake is due to the thresholding, where for example the correct elemental content is identified with high confidence, but plausible alternative elements also end up above the threshold. The tungsten example in Fig. 6 shows that the NN correctly identified tungsten with a significantly higher confidence than rhenium. Yet since the rhenium confidence exceeds the threshold, this kind of prediction is penalized by the EMR. It must be noted that the NN itself has no intrinsic knowledge of any threshold and that thresholding is a form of post-processing that can be replaced, improved or skipped depending on the application and preference of the user.

### Elemental mapping

There are two distinct ways the presented NN can be used in order to fully automate elemental mapping in a spectrum image (SI). One method includes directly mapping the network’s predictions—either before or after thresholding—for each individual spectrum in the SI. The alternative method is using the globally predicted elemental content as input for model-based quantification. In model-based quantification^1,34^, a linear combination of calculated EELS cross sections^22^—usually first convolved with the experimental low-loss spectrum—and a linearized power law background model^45^ are fitted to the spectrum.

**Figure 7:**
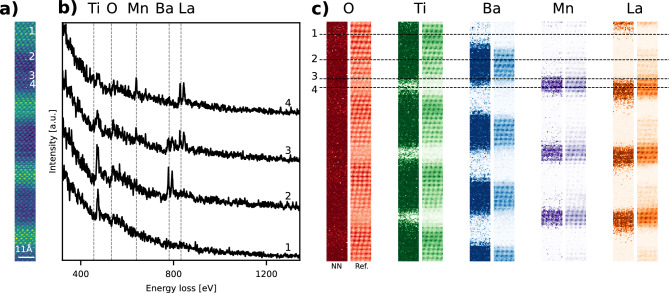
(**a**) Summed intensity of each spectrum in a SI of a ${\text{LaMnO}}_{3}/{\text{BaTiO}}_{3}/{\text{SrTiO}}_{3}$ superlattice. (**b**) Spectra corresponding to marked probe positions. Vertical dashed lines show expected onset energies. (**c**) Mapping results: NN shows the probability of presence as predicted by the ensemble network, Ref. shows the result of a model-based quantification for comparison.

The NN’s predictions on many spectra will likely result in some false positive occurrences in very small concentrations, especially if the spectra are noisy. A selection criterion that only considers elements that are detected in at least 1% of the spectra is preimposed. Figure 7a and b summarize the SI of a ${{\text{LaMnO}}_{3}/{\text{BaTiO}}_{3}/{\text{SrTiO}}_{3}}$ superlattice sample that has been extensively described by Chen et al.^46^. A prediction by the ensemble network on all 7826 spectra in this SI takes approximately one minute on a standard desktop computer with an NVIDIA GeForce RTX 3060 GPU. Given the preimposed selection criterion, the detected elements equal the spectra’s content. The measured energy loss region does not encompass the strontium edges. Probe positions one, two and four are respectively in the ${\mathrm{{SrTiO}}}_{3}$, ${\text{BaTiO}}_{3}$ and ${\text{LaMnO}}_{3}$ region while probe position three is in the transition region between ${{\text{BaTiO}}_{3}}$ and ${\text{LaMnO}}_{3}$. Figure 7c shows the predictions by the ensemble NN and the results of a model-based quantification for comparison. The NN clearly predicts a superlattice structure that matches visual inspection and model-based quantification. Model-based quantification measures some La presence in the ${\text{BaTiO}}_{3}$ region because it cannot perfectly distinguish the overlapping Ba and La edges.

## Conclusion

In this work, a computer generated core-loss EELS dataset, which is useful for training neural networks that generalize effectively to experimental data, is presented. The dataset represents 107 distinct core-loss edges through all 80 elements from Be up to Bi. The use of generic fine structures and low-loss regions result in sufficiently realistic spectra while keeping computational cost limited. Zero padding of the spectra allows resulting models to be applied for experimental spectra measuring virtually any energy loss range within 75 to 3071 eV. We use the data to train a series of neural networks with the task to identify all elements in a given EELS spectrum. Out of a series of compared architectures, the U-Net and the vision transformer presented the best performance when applied to experimental data. Multiple U-Nets and vision transformers are combined in an ensemble network to further increase performance up to a simultaneous precision and recall of both 88%. The application potential is demonstrated by performing fully automated elemental mapping in a ${{\text{LaMnO}}_{3}/{\text{BaTiO}}_{3}/{\text{SrTiO}}_{3}}$ superlattice sample. This work shows the potential for rapid element identification with neural networks and shows their strength in creating input parameters for a model-based quantification process. This combination can form the basis of an entirely unsupervised quantification workflow which is urgently needed to cope with the ever increasing amounts of data that are generated in modern STEM EELS experiments. At the same time they offer the potential to remove the dependency on tuning parameters that inevitably lead to experimenters bias and reproducibility issues that can plague EELS quantification methods.

### Supplementary Information

Supplementary Figures.

## Data Availability

The simulated dataset and trained neural networks are made publicly available on Zenodo at 10.5281/zenodo.8004912.

## Code availability

Code on how to build and use the neural networks is made publicly available on GitHub at arnoannys/EELS_ID.
