# Supplementary material for: Deep learning for automated materials characterisation in core-loss electron energy loss spectroscopy
Source: Sci Rep. 2023 Aug 22;13:13724. doi: 10.1038/s41598-023-40943-7 (PMC10444881; doi:10.1038/s41598-023-40943-7)
Supplement: Supplementary file 1 — Supplementary Figures. [file 41598_2023_40943_MOESM1_ESM.pdf]

Supplementary figures

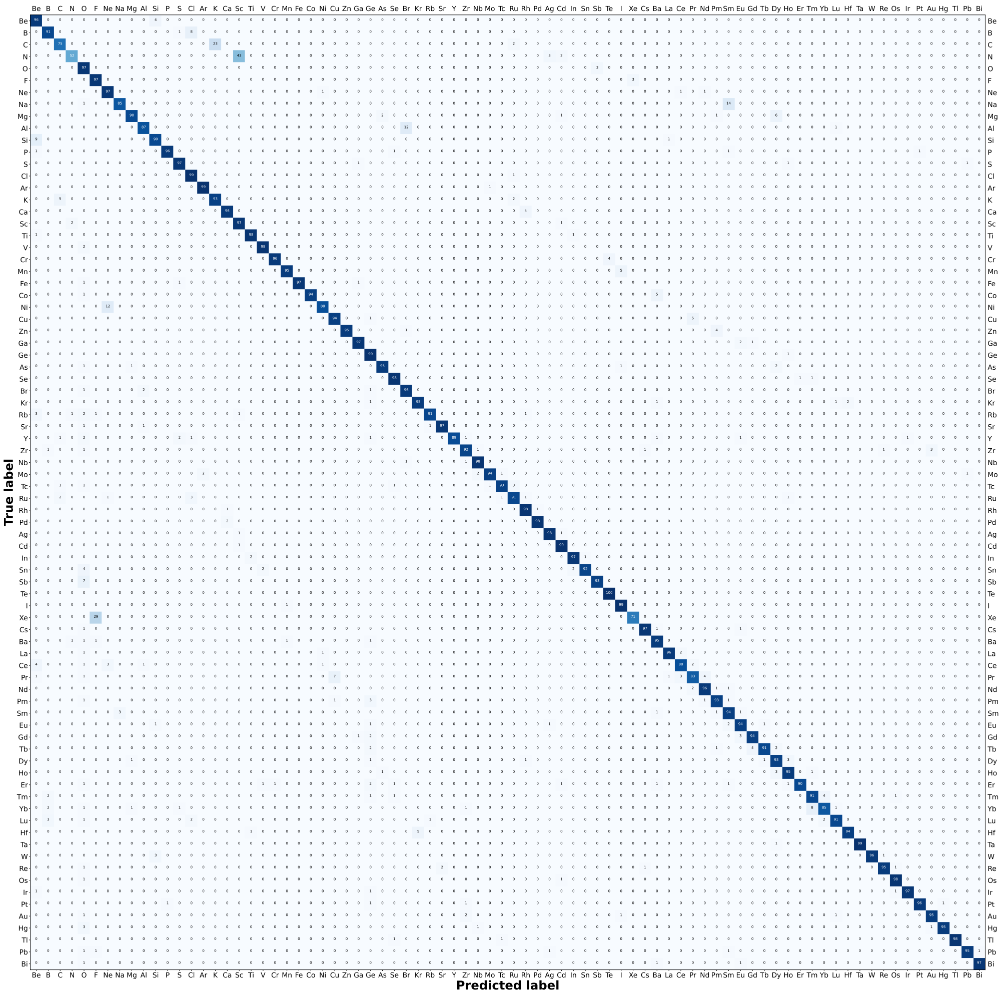

Figure 8. Confusion matrix of the 2×ViT+3×U-Net ensemble

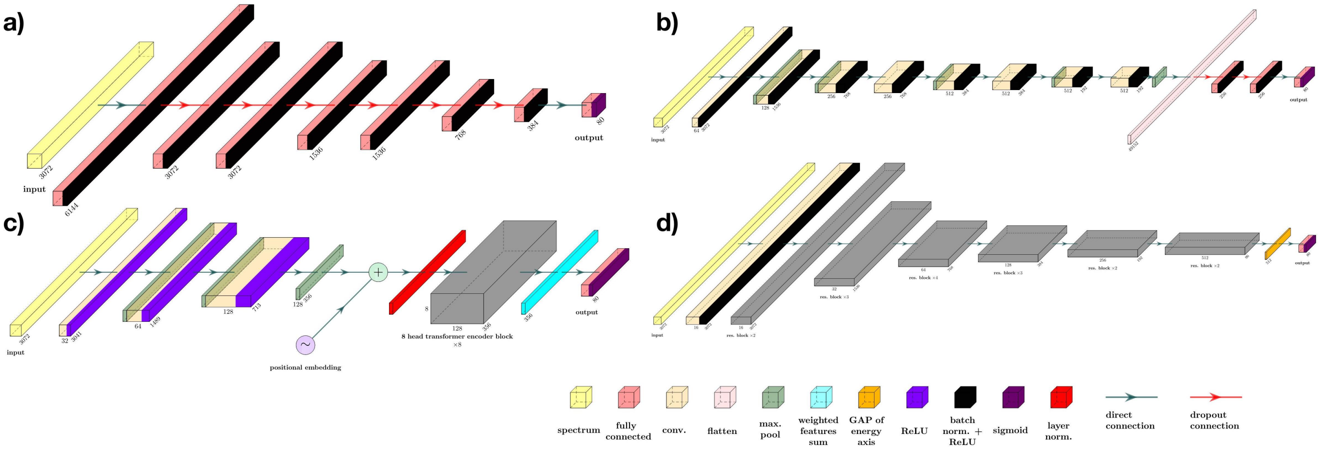

Figure 9. Remaining schematics of compared neural network architectures. **a)** Multilayer perceptron **b)** Convolutional neural network **c)** Compact convolutional transformer **d)** Residual neural network
